# Simian Foamy Virus (SFV) infection from multiple monkey species in women from the Democratic Republic of Congo

**DOI:** 10.1186/1742-4690-8-S1-A233

**Published:** 2011-06-06

**Authors:** William M Switzer, Steve Ahuka-Mundeke, Shaohua Tang, Anupama Shankar, Nathan D Wolfe, Walid Heneine, Martine Peeters, Ahidjo Ayouba, Prime Mulembakani, Anne W Rimoin

**Affiliations:** 1 Division OF HIV/AIDS Prevention, CDC, Atlanta, Georgia, 30333, USA; 2 Institut de Recherche Pour le Développement, Montpellier, France; 3 Global Viral Forecasting Initiative, San Francisco, CA, 94104, USA; 4 Kinshasa School of Public Health, Kinshasa, DRC; 5 University of California at Los Angeles, Los Angeles, CA, 90095, USA

## Background

The recognition that HIV originated from nonhuman primates (NHP) heightens concerns associated with transmission of simian retroviruses to humans. Although recent studies document frequent infection with simian foamy virus (SFV) among primate hunters in Cameroon and primate handlers in North America, little is known about the spread and geographic distribution of SFV outside these populations.

## Materials and methods

We tested 3467 plasma samples collected in 2007 in a population-based survey in 15 rural villages in central Democratic Republic of Congo (DRC). Participants completed a questionnaire about exposure to NHPs. Plasma samples were screened for SFV antibodies using a new Luminex-based EIA; reactive specimens were also tested by a second EIA utilizing recombinant SFV Gag proteins. Specimens dually reactive in both EIAS were then tested using a Western blot (WB) assay that can detect monkey and ape-type SFV reactivity. PCR amplification of DNA from buffy coats and phylogenetic analysis was used to define the NHP species origin of SFV.

## Results

54 (1.6%) plasma samples were dually EIA reactive and 25 (0.72%) were WB positive. Buffy coats were available for PCR testing from 21 persons with WB positive or indeterminate results. SFV polymerase and LTR sequences were amplified from the DNA of three women 23, 50, and 57 years old. Phylogenetic analysis showed novel SFV infection in two women originating from Angolan Colobus (Colobus angolensis) and in one woman from a red-tailed guenon (C. ascanius), viral variants not previously seen in humans. Both monkeys are native to DRC. Two of these women reported exposure to NHPs via butchering and cooking, and specifically to Angolan Colobus or red-tailed guenons. One woman did not report exposure to NHPs. These women lived in three separate villages, suggesting a wide and potentially diverse distribution of SFV infections across DRC. The younger woman reported a history of monkey pox-like illness. All these women were married and had at least three children but specimens were not available from close contacts for testing. Animal exposure data were available for 3034 persons; 2413 (79.3%) reported NHP exposure.

## Conclusions

Our study documents SFV infection in the DRC for the first time and identifies human infection with new SFVs from Colobus and red-tailed monkeys. Our findings suggest that SFV is prevalent across Africa in persons exposed to NHPs inhabiting a specific region. Our results highlight the importance of determining if SFVs are transmitted from person-to-person and can cause disease. SFV infection of humans exposed to NHPs also highlights the increased potential for zoonotic transmission of other simian retroviruses with known pathogenic consequences.

